# A Micromechanics-Based Anisotropic Constitutive Model for Sand Incorporating the True Stress Tensor

**DOI:** 10.3390/ma19020323

**Published:** 2026-01-13

**Authors:** Pengqiang Yu, Hexige Baoyin, Kejia Wu, Haibin Yang

**Affiliations:** 1 Department of Civil Engineering, University of Science and Technology Beijing, Beijing 100083, China; h_baoyin@xs.ustb.edu.cn (H.B.); d202110020@xs.ustb.edu.cn (K.W.); d202540012@xs.ustb.edu.cn (H.Y.); 2 China Railway Construction (Tianjin) Rail Transit Investment Development Co., Ltd., Tianjin 300110, China

**Keywords:** granular micromechanics, sand, anisotropy, constitutive model, true stress, granular materials

## Abstract

To elucidate the micromechanical origins of the macroscopic anisotropic behavior of granular materials, this study develops a micromechanically based elastoplastic constitutive model for sand. First, anchored in the static equilibrium hypothesis and granular micromechanics theory, a true stress tensor is introduced to characterize the authentic inter-particle contact forces. Serving as a coupled variable of the macroscopic stress and the microscopic fabric tensor, this formulation not only quantifies the directional distribution of the contact network but also enables the mapping of anisotropic yielding and deformation analyses into an equivalent isotropic true stress space. Subsequently, a comprehensive constitutive framework is established by integrating critical state theory, an anisotropic fabric evolution law, and an energy-based stress–dilatancy relationship that explicitly accounts for the evolution mechanism of the microscopic coordination number. The physical interpretation, calibration procedure, and sensitivity analysis of the model parameters are also presented. The predictive capability of the model is rigorously validated against conventional triaxial tests on Ottawa sand, true triaxial numerical simulations, and experimental data for Toyoura sand with inherent anisotropy. The comparisons demonstrate that the model accurately captures not only the stress–strain response and volumetric deformation under conventional loading but also the strength dependency on loading direction and mechanical characteristics under complex stress paths, substantiating the validity and universality of the proposed micromechanical approach.

## 1. Introduction

The macroscopic mechanical properties of granular materials, such as sand, are fundamentally governed by particle arrangements and contact interactions at the microscopic scale. Due to variations in depositional environments and subsequent loading histories, sand inevitably exhibits pronounced fabric anisotropy. Generally, this anisotropy is categorized into two distinct types: inherent anisotropy [1] and stress-induced anisotropy [2]. Inherent anisotropy originates from the preferred orientation of particles or the spatial distribution of contacts established during the natural deposition process. Conversely, stress-induced anisotropy arises from the evolution of the internal structure driven by particle sliding, separation, rearrangement, and even crushing under external loading. Since soil anisotropy significantly impacts strength, deformation, and the consequent stability of geotechnical structures, the development of precise anisotropic constitutive models has long been a pivotal research focus in soil mechanics.

To quantitatively characterize the anisotropic mechanics of sand, existing phenomenological approaches can be generally classified into two distinct categories. The first category extends classical isotropic models by rendering key constitutive parameters as functions of the loading orientation, thereby explicitly accounting for the directional dependence of material behavior. For instance, Pietruszczak and Mroz [3,4] generalized the Mohr-Coulomb criterion by treating the friction angle and cohesion as variables dependent on the loading direction relative to the depositional plane. Similarly, Liu and Indraratna [5] proposed an anisotropic Spatially Mobilized Plane (SMP) model [6] by introducing a direction-dependent reduction factor. Analogous parameter-modification strategies have also been widely adopted in the literature [7,8,9]. The second category adopts a more intrinsic approach by incorporating a fabric tensor or a modified stress variable to represent microstructural influences. In this context, Li and Dafalias [10] established a general framework for anisotropic critical state soil mechanics by formulating a state variable derived from the joint invariants of stress and fabric tensors. Alternatively, Yao et al. [11] developed the anisotropic unified hardening model through the implementation of a transformed stress tensor concept. In addition to these invariant-based approaches, the multilaminate framework has been revitalized to address anisotropy. For instance, recent studies [12] have developed state-dependent multilaminate constitutive models that introduce a deviatoric fabric tensor to describe the initial microstructure, employing a micro-level evolution rule to capture the influence of principal stress rotation and induced anisotropy on material behavior. Furthermore, Bayraktaroglu et al. [13] proposed an anisotropic semi-micromechanical cyclic model capable of capturing complex cyclic behaviors.

Beyond the classical elastoplastic framework, advanced hypoplastic constitutive models have also been developed to capture anisotropic behavior without explicitly defining yield surfaces. Notably, Veiskarami et al. [14] proposed a rational hypoplastic equation incorporating a micro-fabric tensor to predict the mechanical response of anisotropic granular materials, and Yu et al. [15] further extended this approach to model the anisotropic behavior of soil-structure contact surfaces considering roughness effects.

Despite their efficacy in reproducing specific experimental observations, these phenomenological models exhibit intrinsic limitations. Primarily, they rely on ad hoc empirical correlations to link anisotropic variables with macroscopic state parameters, thereby lacking a rigorous physical basis. As a result, they fail to provide mechanistic insight into the micromechanical origins of the material response. Furthermore, the need for additional parameters to capture anisotropic evolution complicates model calibration and renders the physical interpretation of these parameters ambiguous.

Micromechanical modeling provides a robust framework to circumvent the limitations of these phenomenological approaches [16]. Rather than explicitly tracking discrete particle trajectories as in the Discrete Element Method (DEM) [17], these methods employ statistical homogenization techniques over a Representative Volume Element (RVE) to rationally upscale micro-scale contact behaviors to macro-scale constitutive relations. Duffy and Mindlin [18] pioneered this field by analyzing the elastic stress-strain relationship of regularly packed spheres. Subsequent research has achieved significant milestones: Chang and Hicher [19] developed a micromechanical elastoplastic framework, known as the CH model, by explicitly incorporating inter-particle sliding and dilatancy mechanisms; Liu and Chang [20] established a linkage between the macroscopic phenomenological elastoplastic framework and the micromechanical CH model, elucidating the correlation between macroscopic and microscopic parameters. Recent extensions of this framework include the work of Tian et al. [21], who combined the CH model with Cosserat continuum theory to account for particle rotation and scale effects, and Wang et al. [22], who explicitly integrated critical state theory into the micromechanical model to enhance its capability in predicting density-dependent fabric evolution. Nicot and Darve [23] introduced the H-model, which utilizes hexagonal particle clusters to capture complex topological deformations [24]. This approach was recently generalized by Miot et al. [25], who developed a 3D constitutive strategy based on the H-model to describe the mechanics of densely packed snow across different scales. In addition, a variety of other micromechanical models have been developed based on similar statistical frameworks [26,27]. More recently, Yu and Liu [28,29] further advanced the field by considering the local fluctuations of particle kinematics, proposing a micromorphic constitutive framework based on micromechanical analysis.

In parallel with these theoretical developments, data-driven approaches leveraging machine learning have emerged as a powerful tool for constitutive modeling. Xiong et al. [30] proposed a physics-informed data-driven model that explicitly considers multiscale particle morphology, while Li and Wang [31] utilized Recurrent Neural Networks (RNNs) to capture the path-dependent constitutive behavior of idealized granular soils, offering a new pathway to bypass complex mathematical formulations.

Despite these advancements, existing micromechanical models face unresolved challenges when applied to strongly anisotropic sands. Specifically, a rigorous definition of the true stress tensor under anisotropic conditions and its intrinsic relationship with macroscopic failure criteria remains underexplored. Moreover, the dynamic evolution of the coordination number during shearing is generally ignored in existing models. This omission compromises the predictive accuracy, particularly concerning stiffness degradation and strain-softening behaviors.

To bridge these gaps and elucidate the micromechanical origins of macroscopic anisotropy, this study develops a micromechanically based elastoplastic constitutive model for sand. The novelties of this work are highlighted as follows: First, anchored in the static equilibrium hypothesis and granular micromechanics, a true stress tensor is rigorously proposed to characterize the authentic inter-particle contact forces. Second, a micromechanics-based strength criterion is established within this true stress space. Unlike conventional criteria based on the macroscopic nominal stress tensor, this new formulation filters out the influence of geometric fabric anisotropy, thereby demonstrating the advantage of recovering a unique, direction-independent strength surface. Third, an energy-based stress–dilatancy relationship is derived via micromechanical analysis, which explicitly accounts for the evolution mechanism of the contact number during shear. Finally, by synthesizing the true stress-based strength criterion and the dilatancy rule with anisotropic fabric evolution and hardening laws, a comprehensive micromechanical constitutive model is formulated. Subsequently, we elucidate the physical interpretation of the model parameters and outline a systematic calibration procedure. Finally, the validity of the proposed framework is demonstrated through comprehensive comparisons with laboratory experiments and numerical simulations, highlighting its efficacy in capturing the complex mechanical response of anisotropic granular materials.

## 2. The True Stress Tensor in Anisotropic Sand

### 2.1. Definition of the Fabric Tensor

As a typical granular material used in geotechnical engineering, the macroscopic mechanical response of sand, such as strength and deformation, is fundamentally governed by the arrangement of particles and their contact networks at the mesoscale. To quantitatively characterize the directional nature of this microstructure, Oda [32] introduced the concept of the fabric tensor. In the analysis of granular assemblies, this tensor is conventionally defined based on the statistical distribution of contact normals ($n_{i}$):

(1) $$F_{ij}=\frac{1}{N}\sum_{c=1}^{N}n_{i}^{c}n_{j}^{c}$$

where *N* denotes the total number of contacts within the Representative Volume Element (RVE), and $n_{i}^{c}$ represents the component of the unit normal vector at the *c*-th contact point. The fabric tensor defined in this manner is a dimensionless, symmetric second-order tensor with a trace of unity (trF=1).

Based on the spectral decomposition of the aforementioned statistical expression, the fabric tensor can be generally expressed in the principal coordinate system ($x_{1},x_{2},x_{3}$) as the following matrix:

(2) $$F_{ij}=\left(\begin{array}{ccc} \frac{1}{3}+a_{1} & 0 & 0\\ 0 & \frac{1}{3}-\frac{a_{1}}{2}+a_{2} & 0\\ 0 & 0 & \frac{1}{3}-\frac{a_{1}}{2}-a_{2} \end{array}\right)$$

where $a_{1}$ and $a_{2}$ are scalar anisotropy parameters. Specifically, the material exhibits isotropy when $a_{1}=a_{2}=0$; transverse isotropy when $a_{1}\neq 0$ and $a_{2}=0$; and orthotropy when both $a_{1}$ and $a_{2}$ are non-zero.

### 2.2. Derivation of the True Stress Tensor

**Figure 1:**
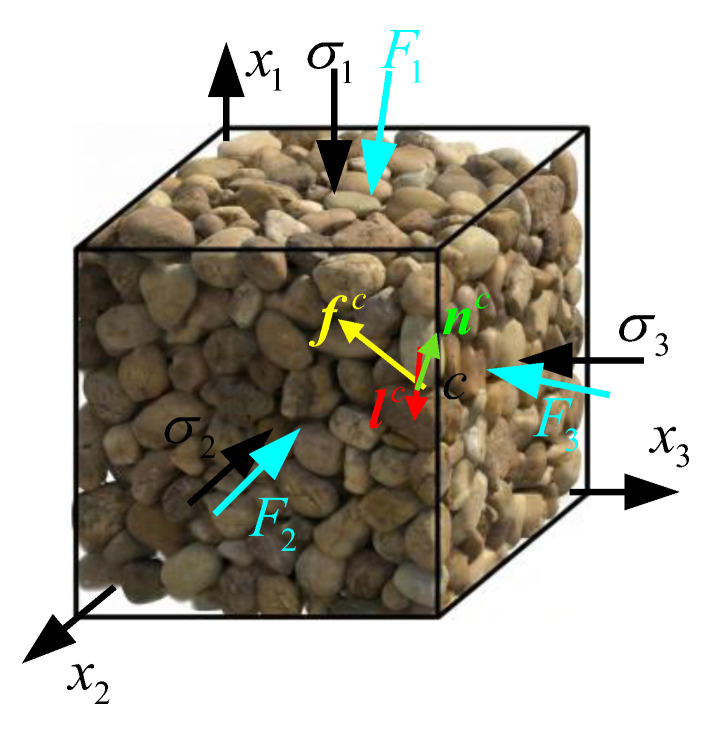
Schematic representation of the micromechanical variables in the RVE.

For granular media, the macroscopic average stress tensor $\sigma_{ij}$ can be derived from contact forces and branch vectors via the Love–Weber formula:

(3) $$\sigma_{ij}=\frac{1}{V}\sum_{c=1}^{N}f_{i}^{c}l_{j}^{c}$$

where *V* is the volume of the RVE, $f_{i}^{c}$ is the contact force vector at the *c*-th contact, and $l_{j}^{c}$ is the branch vector connecting the centers of the two contacting particles, as shown in Figure 1.

The Love–Weber formula (Equation (3)) defines macroscopic stress as the volumetric average of discrete contact forces. However, to construct a predictive constitutive model, one must conversely define how these local contact forces are generated and distributed across the anisotropic granular fabric. To mathematically describe this dependency, it is necessary to postulate a mapping rule linking local geometry to local forces. First, assuming that the sand is composed of spherical particles of average radius $\bar{r}$, the branch vector is collinear with the contact normal, such that $l_{j}^{c}=2\bar{r}n_{j}^{c}$. Second, analogous to the kinematic relationship between macroscopic strain and inter-particle relative displacement [23], we postulate that the microscopic contact force $f_{i}^{c}$ is a linear projection of a second-order tensor $T_{ij}$ onto the branch vector. This tensor $T_{ij}$ is defined as the true stress tensor:

(4) $$f_{i}^{c}=T_{ik}l_{k}^{c}$$

By substituting Equation (4) into the macroscopic stress equation (Equation (3)), we obtain:

(5) $$\sigma_{ij}=\frac{1}{V}\sum_{c=1}^{N}\left(T_{ik}l_{k}^{c}\right)l_{j}^{c}=\frac{1}{V}T_{ik}\sum_{c=1}^{N}l_{k}^{c}l_{j}^{c}$$

Substituting the geometric condition $l_{k}^{c}=2\bar{r}n_{k}^{c}$ into the summation term yields:

(6) $$\sum_{c=1}^{N}l_{k}^{c}l_{j}^{c}=\sum_{c=1}^{N}\left(2\bar{r}n_{k}^{c}\right)\left(2\bar{r}n_{j}^{c}\right)=4{\bar{r}}^{2}\sum_{c=1}^{N}n_{k}^{c}n_{j}^{c}$$

Recalling the definition of the fabric tensor, the summation term can be rewritten as:

(7) $$\sum_{c=1}^{N}l_{k}^{c}l_{j}^{c}=4{\bar{r}}^{2}NF_{kj}$$

Consequently, the relationship between the macroscopic stress tensor and the True Stress Tensor is derived as:

(8) $$\sigma_{ij}=\frac{4{\bar{r}}^{2}N}{V}T_{ik}F_{kj}$$

Letting the scalar structural coefficient $\xi =\frac{V}{4{\bar{r}}^{2}N}$, and applying tensor inversion, we obtain the explicit expression for the true stress tensor $T_{ij}$:

(9) $$T_{ij}=\xi \sigma_{ik}F_{kj}^{-1}$$

Equation (9) elucidates the fundamental distinction between the macroscopic phenomenological stress and the true stress. This relationship offers significant physical insights into the mechanics of anisotropic granular media: (1) The macroscopic nominal stress $\sigma_{ij}$ is inherently a coupled variable, amalgamating the material’s mechanical state with its geometric fabric. In anisotropic sands, due to the non-uniformity of the fabric $F_{ij}$, the distribution of microscopic contact forces remains non-uniform even under isotropic macroscopic loading. The true stress $T_{ij}$, by incorporating the inverse of the fabric tensor $F_{kj}^{-1}$, effectively filters out the geometric anisotropy of the particle arrangement, thereby recovering the authentic stress intensity acting on the solid skeleton. (2) Macroscopic failure in granular materials stems from inter-particle sliding and rolling, both of which are strictly governed by local contact forces. As the true stress $T_{ij}$ directly determines the magnitude and orientation of these forces, it serves as the fundamental variable controlling yield and failure. Consequently, formulating failure criteria within the true stress space theoretically eliminates the directional dependence of strength parameters, leading to a physically clearer constitutive model.

It is important to state that, to obtain a closed-form analytical relationship between the macroscopic nominal stress and the true stress, the granular assembly is idealized as a packing of rigid spherical particles. Consequently, the branch vector connecting particle centers is assumed to be collinear with the contact normal vector. While natural sands possess angular morphologies that violate this collinearity, the mathematical structure derived from this idealization provides a robust baseline. The effects of particle angularity and surface roughness in real sands are not explicitly modeled geometrically but are implicitly captured through the calibration of macroscopic material constants in practice.

## 3. Formulation of the Micromechanical Constitutive Model

Building upon the true stress tensor formulation derived in the preceding section and incorporating the framework of critical state theory, a micromechanical-based elastoplastic constitutive model for anisotropic granular materials is established.

### 3.1. Elastic Behaviors

The total strain increment is assumed to decompose additively into elastic and plastic components. Consequently, the volumetric and deviatoric strain increments ($d\varepsilon_{v}$ and $d\varepsilon_{q}$) are expressed as:

(10) $$\left\{\begin{array}{cc} d\varepsilon_{v} & =d\varepsilon_{v}^{e}+d\varepsilon_{v}^{p}\\ d\varepsilon_{q} & =d\varepsilon_{q}^{e}+d\varepsilon_{q}^{p} \end{array}\right.$$

The elastic response is governed by the generalized Hooke’s Law. In incremental form, the relationship between stress increments and elastic strain increments is given by:

(11) $$\left\{\begin{array}{cc} dp & =Kd\varepsilon_{v}^{e}\\ dq & =3Gd\varepsilon_{q}^{e} \end{array}\right.$$

where $p=\frac{1}{3}\sigma_{ii}$ and $q=\sqrt{\frac{3}{2}\sigma_{ij}^{\prime }\sigma_{ij}^{\prime }}$ ($\sigma_{ij}^{\prime }$ denotes the deviatoric stress tensor) denote the mean stress and deviatoric stress, respectively; *K* and *G* represent the elastic bulk modulus and shear modulus. To capture the stress-level and density dependence of soil stiffness, the shear modulus *G* is evaluated using the empirical formulation proposed by Hardin and Drnevich [33]:

(12) $$G=G_{0}\frac{{\left(c_{g}-e\right)}^{2}}{1+e}{\left(\frac{p}{p_{ref}}\right)}^{0.5}$$

where $p_{ref}$ is a reference pressure (taken as 1kPa); $G_{0}$ and $c_{g}$ are material constants; and *e* represents the current void ratio, which evolves from the initial void ratio $e_{0}$ according to the relation $e=e_{0}-\left(1+e_{0}\right)\varepsilon_{v}$.

Assuming a constant Poisson’s ratio μ, the bulk modulus *K* is related to the shear modulus via the classical elasticity relationship:

(13) $$K=G\frac{2(1+\mu )}{3(1-2\mu )}$$

### 3.2. Yield Surface

In Section 2.1, based on the projection rule [34], it was derived that the particle contact force can be characterized as the projection of the true stress tensor along the direction of the branch vector, expressed as:

(14) $$f_{i}=2\bar{r}T_{ij}n_{j}$$

Consequently, the three components of the contact force vector in three-dimensional space are derived as:

(15) $$\left\{\begin{array}{cc} f_{x} & =\frac{V}{2\bar{r}N}\frac{\sigma_{xx}}{F_{xx}}n_{x}\\ f_{y} & =\frac{V}{2\bar{r}N}\frac{\sigma_{yy}}{F_{yy}}n_{y}\\ f_{z} & =\frac{V}{2\bar{r}N}\frac{\sigma_{zz}}{F_{zz}}n_{z} \end{array}\right.$$

Furthermore, the inter-particle normal contact force, $f_{n}$, and tangential contact force, $f_{s}$, are obtained as follows:

(16) $$f_{n}=f_{i}n_{i}=\frac{V}{2\bar{r}N}\left(\frac{\sigma_{xx}}{F_{xx}}n_{x}^{2}+\frac{\sigma_{yy}}{F_{yy}}n_{y}^{2}+\frac{\sigma_{zz}}{F_{zz}}n_{z}^{2}\right)$$

(17) $$f_{s}=\sqrt{f_{i}^{2}-f_{n}^{2}}=\frac{V}{2\bar{r}N}\sqrt{\left(\frac{\sigma_{xx}}{F_{xx}}-\frac{\sigma_{yy}}{F_{yy}}\right)n_{x}^{2}n_{y}^{2}+\left(\frac{\sigma_{yy}}{F_{yy}}-\frac{\sigma_{zz}}{F_{zz}}\right)n_{y}^{2}n_{z}^{2}+\left(\frac{\sigma_{xx}}{F_{xx}}-\frac{\sigma_{zz}}{F_{zz}}\right)n_{x}^{2}n_{z}^{2}}$$

The ratio of the tangential to the normal contact force is then expressed in terms of the true stress tensor:

(18) $$\frac{f_{s}}{f_{n}}=\frac{\sqrt{{\left(T_{xx}-T_{yy}\right)}^{2}n_{x}^{2}n_{y}^{2}+{\left(T_{zz}-T_{yy}\right)}^{2}n_{z}^{2}n_{y}^{2}+{\left(T_{xx}-T_{zz}\right)}^{2}n_{x}^{2}n_{z}^{2}}}{T_{xx}n_{x}^{2}+T_{yy}n_{y}^{2}+T_{zz}n_{z}^{2}}$$

This formulation effectively maps the generalized Coulomb criterion into the true stress space, allowing the anisotropic yielding behavior to be analyzed within an equivalent isotropic framework. To identify the critical state of stress yielding, the concept of a generalized spatial mobilized plane is introduced. The orientation of this potential sliding plane is governed by the true stress components:

(19) $${\left(\frac{n_{i}}{n_{j}}\right)}^{2}={\left(\frac{T_{j}}{T_{i}}\right)}^{m}$$

The equation above defines the orientation where the ratio of shear stress to normal stress (i.e., the mobilized friction) is maximized within the true stress space. The parameter *m* governs the sensitivity of the sliding plane orientation to the stress anisotropy. Notably, for isotropic materials where m=1, this relationship degenerates to the orientation of the spatial mobilized plane defined by the classical Matsuoka-Nakai criterion.

Adopting a statistical homogenization approach, it is postulated that while microscopic sliding is progressive, macroscopic yielding occurs when the averaged contact force ratio on this potential sliding plane reaches a critical threshold [35]. Substituting the direction cosines into the contact force ratio formulation yields the yield surface equation:

(20) $$f\left(T_{1},T_{2},T_{3},H_{T}\right)=\frac{\sqrt{\frac{{\left(T_{1}-T_{2}\right)}^{2}}{T_{1}^{m}T_{2}^{m}}+\frac{{\left(T_{1}-T_{3}\right)}^{2}}{T_{1}^{m}T_{3}^{m}}+\frac{{\left(T_{2}-T_{3}\right)}^{2}}{T_{2}^{m}T_{3}^{m}}}}{T_{1}^{1-m}+T_{2}^{1-m}+T_{3}^{1-m}}-H_{T}=0$$

where $T_{1},T_{2},T_{3}$ denote the principal true stresses, and $H_{T}$ is the hardening parameter representing the peak contact force ratio at the current state.

**Figure 2:**
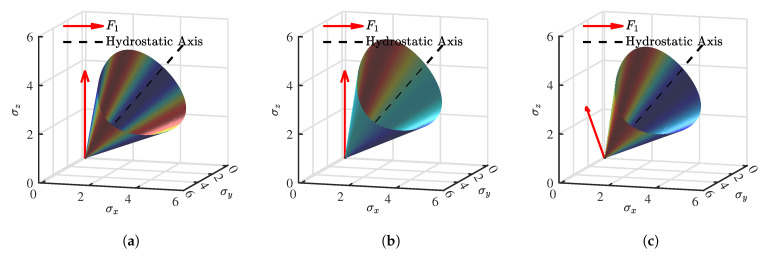
Visualization of the yield surfaces in principal stress space under different fabric conditions: (**a**) isotropic case; (**b**) coaxial anisotropic case; (**c**) non-coaxial anisotropic case.

To visually elucidate the influence of fabric anisotropy on the yielding behavior, the yield surfaces in the principal stress space are plotted in Figure 2. In these visualizations, the jet colormap is employed to represent the magnitude of the deviatoric stress, with the color gradient from blue to red indicating values transitioning from low to high. Three distinct fabric states are considered: (a) Isotropic state ($F_{11}=F_{22}=F_{33}=1/3$): As shown in Figure 2a, the yield surface exhibits a standard rounded triangular shape with three-fold symmetry around the hydrostatic axis, identical to the isotropic Matsuoka-Nakai criterion. (b) Coaxial Anisotropic state ($F_{11}=0.36,F_{22}=F_{33}=0.32$): When the major principal axis of the fabric coincides with the major principal stress axis, the yield surface elongates along the direction of the strong fabric ($F_{11}$), as depicted in Figure 2b. This reflects the enhanced shear resistance in the direction of particle deposition. (c) Non-coaxial Anisotropic state ($F_{11}=0.36,F_{22}=F_{33}=0.32$, with a 45° angle between $F_{11}$ and $\sigma_{z}$): When the principal axes of stress and fabric are non-coaxial (rotated by 45°), the yield surface undergoes a distinct rotation and distortion relative to the stress axes (Figure 2c). This comparison highlights the model’s capability to capture the direction-dependent strength of sand by mapping the isotropic yield criterion from the true stress space back to the macroscopic nominal stress space.

### 3.3. Stress–Dilatancy Relationship

From a micromechanical perspective, the total increment of internal energy dissipation, dψ, within a granular assembly during shearing comprises two components: frictional dissipation arising from inter-particle sliding and structural dissipation resulting from the evolution of contact topology (creation and disruption of contacts). This can be approximated as:

(21) dψ=Ndw+wdN

where *N* denotes the total number of contacts; *w* represents the average dissipated energy per contact; and dN and dw denote the increments of the contact number and the average energy dissipation per contact, respectively. According to the derivation by Liu et al. [36], the average energy dissipation increment at a single contact, dw, is formulated as:

(22) $$dw={\bar{f}}_{s}\cdot d{\bar{u}}_{s}=\frac{4{\bar{r}}^{2}}{5}\left(\frac{N_{s}}{N}\right)q_{T}d\varepsilon_{q}\sqrt{N_{T}N_{\dot{\varepsilon }}}$$

where ${\bar{f}}_{s}$ is the average tangential contact force; ${\bar{u}}_{s}$ is the average tangential sliding displacement; $N_{s}$ is the number of sliding contacts; $q_{T}$ represents the generalized true deviatoric stress; and $N_{T}$ and $N_{\dot{\varepsilon }}$ are complex statistical functions characterizing the contact force distribution and inter-particle displacement, respectively (see [36] for details).

To capture the evolution of the micro-topology during shear, the increment of the contact number, dN, is postulated to follow the evolution law:

(23) $$dN=N_{0}k\left[1-\left(\frac{\varepsilon_{q}}{\chi +\varepsilon_{q}}\right){\left(\frac{e}{e_{cr}}\right)}^{-\alpha }\right]d\varepsilon_{q}$$

where α, χ, and *k* are model constants; $N_{0}$ is the initial contact number at the onset of shearing; and *e* and $e_{cr}$ denote the current and critical void ratios, respectively.

To strictly justify the physical validity of the proposed evolution law for the coordination number (Equation (23)), a series of 3D DEM simulations were conducted using the commercial code PFC3D 5.0. A cylindrical numerical specimen was generated, comprising approximately 8000 spherical particles with sizes following a uniform distribution from 0.2 mm to 0.5 mm. The inter-particle mechanical behavior was governed by the standard linear contact model, with microscopic parameters calibrated to represent generic quartz sand. Specifically, the particle density was set to 2600 kg/$m^{3}$, the inter-particle friction coefficient to 0.5, and the normal and shear contact stiffnesses were assigned values of $1\times 10^{5}$ N/m and $0.8\times 10^{5}$ N/m, respectively. To investigate the microstructural evolution under varying conditions, two distinct groups of samples were prepared: loose specimens with an initial void ratio of approximately 0.805, and dense specimens with an initial void ratio of approximately 0.638. These samples were isotropically consolidated to three different confining pressures ($\sigma_{3}=200,\;500,$ and 800 kPa) and subsequently subjected to standard drained triaxial compression tests. Throughout the shearing process, the average contact number *N* was monitored in real-time to record the variation of the contact network structure.

**Figure 3:**
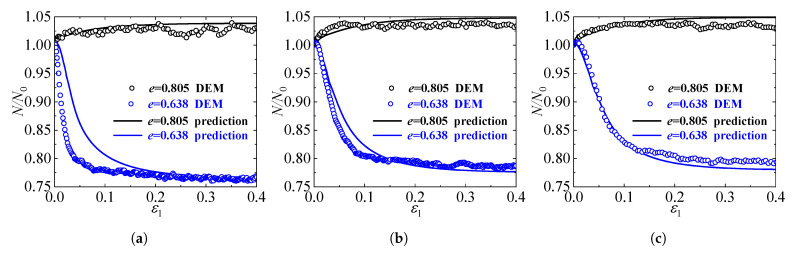
Comparisons of the normalized contact number evolution between model predictions and DEM simulations for loose and dense specimens under confining pressures of (**a**) 200 kPa, (**b**) 500 kPa, and (**c**) 800 kPa.

Figure 3 presents the comparisons between the theoretical predictions and the DEM simulation results for the evolution of the contact number under confining pressures of 200, 500, and 800 kPa. The vertical axis represents the normalized contact number $N/N_{0}$, where $N_{0}$ denotes the initial value at the onset of shearing; thus, all curves originate from unity. As illustrated in the figures, the evolution patterns are fundamentally governed by the initial density state. For the dense specimens (e=0.638), the normalized contact number exhibits a sharp, monotonic reduction during the initial shearing phase. This decay is attributed to the significant volumetric dilatancy inherent in dense sands. Conversely, the loose specimens (e=0.805) display a distinct increasing trend in the contact number. This behavior corresponds to the contractive response of loose sand, where the densification of the packing structure facilitates the formation of new contacts. Regardless of the divergent initial trajectories, the contact numbers for both loose and dense samples gradually stabilize as the material approaches the critical state. The satisfactory agreement between the model predictions and the DEM measurements confirms that the proposed evolution law effectively captures the coupling between macroscopic volumetric deformation and the microscopic evolution of the contact network.

Postulating that the macroscopic external work increment equates to the total microscopic internal energy dissipation (i.e., energy conservation):

(24) $$pd\varepsilon_{v}+qd\varepsilon_{q}=Ndw+wdN$$

Substituting the expressions for energy dissipation and contact evolution (Equations (21) and (23)) into the conservation equation yields, after rearrangement, the explicit stress–dilatancy relationship:

(25) $$D=-d\varepsilon_{v}/d\varepsilon_{q}=C\frac{q_{T}}{p}-\frac{q}{p}+\lambda D_{0}$$

where the state variable $\lambda =1-\left[\varepsilon_{q}/\left(\chi +\varepsilon_{q}\right)\right]{\left(e/e_{cr}\right)}^{-\alpha }$ characterizes the rate of contact number variation; $C=4{\bar{r}}^{2}N_{S}\sqrt{N_{T}N_{\dot{\varepsilon }}}/\left(5V\right)$ is a complex function reflecting the microstructural properties and sample volume *V*; and $D_{0}=N_{0}kw/p$ represents the characteristic initial dilatancy parameter, which is assumed to remain constant throughout the loading process in this study.

Given that the direct determination of parameter *C* is complicated by its dependence on microscopic statistical variables, a simplified approach based on critical state theory is proposed. At the critical state, the plastic volumetric strain ceases to evolve (i.e., D=0), and the microstructure reaches a dynamic equilibrium where the net contact number remains unchanged (implying λ→0). Applying these conditions to the dilatancy equation yields the critical value of parameter *C*:

(26) $$C_{cr}={\left(\frac{q}{q_{T}}\right)}_{cr}$$

Since the coordination number is intrinsically linked to the void ratio, and recognizing that *C* incorporates the effect of the sliding contact number $N_{s}$ and density, *C* is postulated to evolve as a state-dependent function of the void ratio *e*:

(27) $$C=C_{cr}{\left(\frac{e}{e_{cr}}\right)}^{\alpha }$$

Finally, substituting Equation (27) into Equation (25) yields the proposed macroscopic stress–dilatancy relationship, which explicitly accounts for the evolution mechanism of the microscopic contact number.

**Figure 4:**
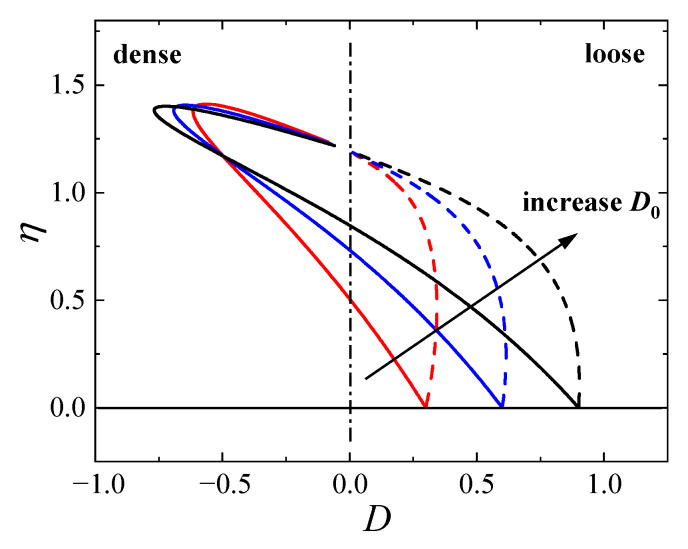
Predicted stress–dilatancy relationships for loose and dense sands with different initial dilatancy parameters $D_{0}$.

The behavior of the derived stress–dilatancy relationship (Equation (25)) is illustrated in Figure 4. The curves depict the evolution of the dilatancy ratio *D* versus the stress ratio η (=q/p) for both loose and dense specimens with varying initial dilatancy parameters $D_{0}$. For dense sand (solid lines), the model predicts a distinct phase transformation behavior: the material initially undergoes volumetric contraction (D>0) followed by significant dilation (D<0) after crossing the phase transformation line (D=0). An increase in the parameter $D_{0}$ enhances the initial contraction tendency. For loose sand (dashed lines), the material exhibits continuous contraction throughout the shearing process, approaching a constant volume state (D=0) at large strains. These trends are consistent with typical experimental observations for granular materials.

### 3.4. Harding Rule

The proposed constitutive model employs a hyperbolic hardening law to describe the plastic evolution of the material, expressed as:

(28) $$H\left(\varepsilon_{q}^{p}\right)=\frac{k_{0}^{p}M\varepsilon_{q}^{p}}{pM+k_{0}^{p}\varepsilon_{q}^{p}}$$

where $k_{0}^{p}$ represents the initial plastic bulk modulus, and *M* denotes the stress ratio at the critical state, defined as $M=6\sin\varphi_{p}/\left(3-\sin\varphi_{p}\right)$. Here, the peak friction angle $\varphi_{p}$ is a state-dependent variable related to the material density, governed by the following relationship:

(29) $$\tan\varphi_{p}={\left(\frac{e_{cr}}{e}\right)}^{m_{\varphi }}\tan\varphi_{cr}$$

where $m_{\varphi }$ is a material parameter reflecting the sensitivity of the granular assembly to its density state.

**Figure 5:**
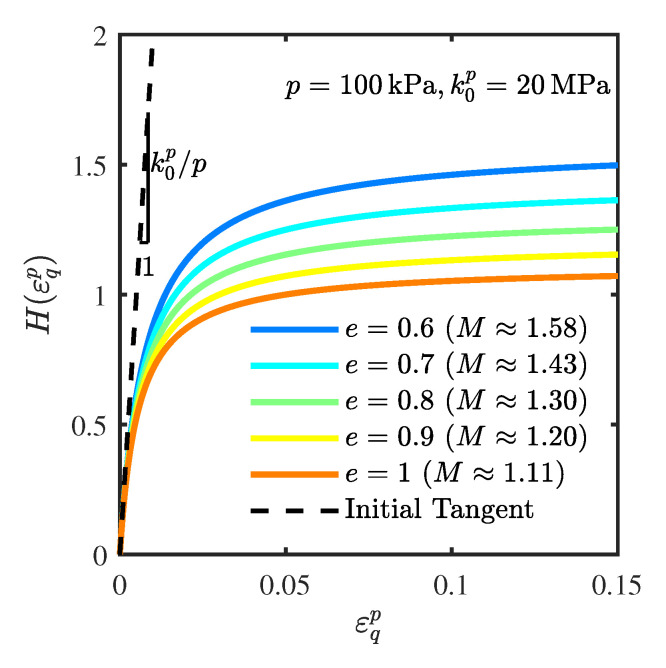
Evolution of hardening curves for specimens with different initial void ratios.

Figure 5 presents the evolution of the mobilized friction resistance (hardening curves) for specimens with different initial void ratios, governed by the proposed hardening law. It can be observed that while the initial slopes of all curves are identical (governed by the normalized initial plastic modulus $k_{0}^{p}/p$), the subsequent evolution characteristics are fundamentally controlled by the initial void ratio. Specifically, the dense specimen (low void ratio) exhibits a stiffer response, where the hardening parameter *H* increases rapidly and reaches a higher magnitude. In contrast, the loose specimen (high void ratio) shows a more gradual response, with *H* increasing slowly and remaining at a lower level. This density-dependent evolution reflects how the initial density constraints the mobilization of frictional resistance.

Based on the hardening function, the ratio of the major to minor principal stresses, $k_{13}$, at the current plastic state can be derived as:

(30) $$k_{13}\left(\varepsilon_{q}^{p}\right)=\frac{\left(2+b\right)H\left(\varepsilon_{q}^{p}\right)+3\sqrt{b^{2}-b+1}}{3\sqrt{b^{2}-b+1}-\left(b+1\right)H\left(\varepsilon_{q}^{p}\right)}$$

where *b* is the intermediate principal stress parameter. Incorporating the fabric evolution equations, the principal stress ratios in the true stress space, $k_{T13}$ and $k_{T23}$, are explicitly formulated as:

(31) $$\begin{array}{cc} k_{T13} & =\frac{T_{1}}{T_{3}}=k_{13}\left(\varepsilon_{q}^{p}\right)\times \left[\frac{F_{33}^{0}+\beta \left(\frac{3}{\left(b+1\right)k_{13}\left(\varepsilon_{q}^{p}\right)+2-b}-1\right)}{F_{11}^{0}+\beta \left(\frac{3k_{13}\left(\varepsilon_{q}^{p}\right)}{\left(b+1\right)k_{13}\left(\varepsilon_{q}^{p}\right)+2-b}-1\right)}\right]\\ k_{T23} & =\frac{T_{2}}{T_{3}}=\left[bk_{13}\left(\varepsilon_{q}^{p}\right)+1-b\right]\times \left[\frac{F_{33}^{0}+\beta \left(\frac{3}{\left(b+1\right)k_{13}\left(\varepsilon_{q}^{p}\right)+2-b}-1\right)}{F_{22}^{0}+\beta \left(\frac{3(bk_{13}\left(\varepsilon_{q}^{p}\right)+1-b)}{\left(b+1\right)k_{13}\left(\varepsilon_{q}^{p}\right)+2-b}-1\right)}\right] \end{array}$$

According to the definition of the yield function, the hardening parameter $H_{T}$, which characterizes the mobilized strength in the true stress space, is expressed in terms of the principal true stress components:

(32) $$H_{T}=\frac{\sqrt{{\left(T_{1}-T_{2}\right)}^{2}T_{3}^{m}+{\left(T_{1}-T_{3}\right)}^{2}T_{2}^{m}+{\left(T_{2}-T_{3}\right)}^{2}T_{1}^{m}}}{T_{1}^{1-\frac{m}{2}}T_{2}^{\frac{m}{2}}T_{3}^{\frac{m}{2}}+T_{2}^{1-\frac{m}{2}}T_{1}^{\frac{m}{2}}T_{3}^{\frac{m}{2}}+T_{3}^{1-\frac{m}{2}}T_{1}^{\frac{m}{2}}T_{2}^{\frac{m}{2}}}$$

By normalizing this equation (dividing both the numerator and denominator by $T_{3}$) and introducing the true stress ratios $k_{T13}$ and $k_{T23}$, the dimensionless form of $H_{T}$ is obtained:

(33) $$H_{T}=\frac{\sqrt{{\left(k_{T13}-k_{T23}\right)}^{2}+{\left(k_{T13}-1\right)}^{2}k_{T23}^{m}+{\left(k_{T23}-1\right)}^{2}k_{T13}^{m}}}{k_{T13}^{1-\frac{m}{2}}k_{T23}^{\frac{m}{2}}+k_{T13}^{\frac{m}{2}}k_{T23}^{1-\frac{m}{2}}+k_{T13}^{\frac{m}{2}}k_{T23}^{\frac{m}{2}}}$$

Substituting Equation (31) into this expression provides the complete computational procedure for determining the hardening parameter $H_{T}$.

### 3.5. Fabric Evolution

To account for fabric evolution during the shearing process, the proposed model adopts the fabric evolution law proposed by Guo and Stolle [37], which is expressed in incremental form as:

(34) $${\dot{F}}_{ij}=\beta \frac{{\dot{\sigma }}_{ij}}{p}$$

Upon integration, the expression for the current fabric tensor is obtained:

(35) $$F_{ij}=\beta \left(\frac{\sigma_{ij}}{p}-\delta_{ij}\right)+F_{ij}{|}_{0}$$

where the parameter β characterizes the sensitivity of fabric evolution to the material’s frictional properties, and $F_{ij}{|}_{0}$ denotes the initial fabric tensor corresponding to the incipient depositional state, $\delta_{ij}$ denotes the Kronecker delta.

### 3.6. Critical State

Within the framework of critical state soil mechanics, the Critical State Line (CSL) in the e−p plane is mathematically characterized by the following exponential formulation:

(36) $$e_{cr}=e_{cr0}\exp\left[-{\left(\frac{p}{h_{cr}}\right)}^{n_{cr}}\right]$$

where $e_{cr0}$ denotes the reference critical void ratio; while $h_{cr}$ and $n_{cr}$ are material parameters governing the geometric shape of the CSL.

### 3.7. Incremental Constitutive Relations

Synthesizing the elastic relationships, yield function, hardening law, dilatancy rule, and fabric evolution established in the preceding sections, the complete incremental elastoplastic constitutive model is formulated. Within this framework, the coupled relationship between total strain increments (volumetric strain increment $d\varepsilon_{v}$ and deviatoric strain increment $d\varepsilon_{q}$) and stress increments (mean effective stress increment dp and deviatoric stress increment dq) is expressed in the compliance matrix form as follows:

(37) $$\left\{\begin{array}{c} d\varepsilon_{v}\\ d\varepsilon_{q} \end{array}\right\}=\left(\begin{array}{cc} C_{11} & C_{12}\\ C_{21} & C_{22} \end{array}\right)\left\{\begin{array}{c} dp\\ dq \end{array}\right\}$$

Here, the elastic strain increments are determined via the generalized Hooke’s law derived previously (Equation (10)). The plastic volumetric strain increment $d\varepsilon_{v}^{p}$ and plastic deviatoric strain increment $d\varepsilon_{q}^{p}$ are derived based on the consistency condition and the flow rule. These plastic components are expressed as:

(38) $$\left\{\begin{array}{cc} d\varepsilon_{v}^{p} & =\frac{\frac{\partial f}{\partial p}dp+\frac{\partial f}{\partial q}dq}{-\frac{\partial f}{\partial H}\frac{\partial H}{\partial \varepsilon_{q}^{p}}}D\\ d\varepsilon_{q}^{p} & =\frac{\frac{\partial f}{\partial p}dp+\frac{\partial f}{\partial q}dq}{-\frac{\partial f}{\partial H}\frac{\partial H}{\partial \varepsilon_{q}^{p}}} \end{array}\right.$$

By superposing the elastic and plastic components, the specific expressions for the elements of the compliance matrix $C_{ij}$ are determined:

(39) $$\left\{\begin{array}{cc} C_{11} & =\frac{1}{K}+\frac{D\frac{\partial f}{\partial p}}{-\frac{\partial f}{\partial H}\frac{\partial H}{\partial \varepsilon_{q}^{p}}}\\ C_{12} & =\frac{D\frac{\partial f}{\partial q}}{-\frac{\partial f}{\partial H}\frac{\partial H}{\partial \varepsilon_{q}^{p}}}\\ C_{21} & =\frac{\frac{\partial f}{\partial p}}{-\frac{\partial f}{\partial H}\frac{\partial H}{\partial \varepsilon_{q}^{p}}}\\ C_{22} & =\frac{1}{3G}+\frac{\frac{\partial f}{\partial q}}{-\frac{\partial f}{\partial H}\frac{\partial H}{\partial \varepsilon_{q}^{p}}} \end{array}\right.$$

It is imperative to note that since the yield function *f* in this model is formulated based on the true stress tensor rather than the conventional stress tensor, the derivation of the partial derivatives (e.g., $\frac{\partial f}{\partial p}$ and $\frac{\partial f}{\partial q}$) requires the rigorous application of the chain rule to map gradients from the true stress space to the conventional stress space.

## 4. Parameter Determination and Discussion

### 4.1. Parameter Determination and Sensitivity Analysis

The proposed constitutive framework incorporates a total of sixteen material parameters. Based on their distinct physical interpretations and functional roles, these parameters are categorized into five groups, as list in Table 1.

Among these, the elasticity and critical state parameters constitute the foundation of classical critical state soil mechanics. Since their calibration procedures and influencing factors have been extensively investigated and documented in existing literature [38], they warrant no detailed elaboration here. Regarding the initialization parameters, the initial fabric components ($F_{11}^{0},F_{22}^{0},F_{33}^{0}$) are determined directly by the degree of inherent anisotropy of the specimen, while the critical state friction angle ($\varphi_{cr}$) is derived from the critical stress ratio. Consequently, the subsequent discussion focuses specifically on the determination and physical significance of the remaining parameters, with particular emphasis on the strength parameters and the plastic deformation (dilatancy) parameters unique to this framework.

(1) Fabric evolution parameter β

The fabric evolution parameter β characterizes the kinetics of microstructural evolution driven by stress variations and is intrinsically linked to the frictional properties of the granular assembly. For frictional granular materials, the macroscopic mobilized friction angle, $\varphi_{m}$, is defined by the principal stress ratio as:

(40) $$\frac{\sigma_{1}}{\sigma_{3}}=\frac{1+\sin\varphi_{m}}{1-\sin\varphi_{m}}$$

Analogously, the microscopic mobilized friction angle, $\varphi_{m}^{\prime }$, defined within the true stress space, is expressed as:

(41) $$\frac{T_{1}}{T_{3}}=\frac{1+\sin\varphi_{m}^{\prime }}{1-\sin\varphi_{m}^{\prime }}$$

In anisotropic granular media, material failure is fundamentally governed by friction mobilization at the contact level. From a micromechanical perspective, yield and inter-particle sliding initiate when the microscopic mobilized friction angle $\varphi_{m}^{\prime }$ reaches the intrinsic inter-particle friction angle $\varphi_{u}$. Consequently, the relationship between fabric anisotropy and the macroscopic stress ratio at failure follows:

(42) $$\frac{F_{11}}{F_{33}}=\frac{1-\sin\varphi_{u}}{1+\sin\varphi_{u}}\frac{\sigma_{1}}{\sigma_{3}}$$

Substituting the fabric evolution law into the above equation and invoking the critical state condition (assuming the initial anisotropy is fully obliterated), the ratio of fabric components at the critical state is given by:

(43) $${\left(\frac{F_{11}}{F_{33}}\right)}_{cr}=\frac{\frac{\beta }{p}{\left(\sigma_{11}\right)}_{cr}+\frac{1}{3}-\beta }{\frac{\beta }{p}{\left(\sigma_{33}\right)}_{cr}+\frac{1}{3}-\beta }=\frac{1-\sin\varphi_{u,cr}}{1+\sin\varphi_{u,cr}}{\left(\frac{\sigma_{1}}{\sigma_{3}}\right)}_{cr}$$

where $\varphi_{u,cr}$ denotes the critical inter-particle friction angle. Reflecting the dynamic equilibrium of microstructural reorganization at the critical state, this parameter is statistically defined as the ratio of the total tangential contact forces to the total normal contact forces within the specimen, expressed as $\tan\varphi_{u,cr}=\frac{\sum {\left(f_{s}^{c}\right)}_{cr}}{\sum {\left(f_{n}^{c}\right)}_{cr}}$ (where $f_{s}^{c}$ and $f_{n}^{c}$ represent the tangential and normal forces at contact *c*). Rearranging the terms yields the explicit formulation for β:

(44) $$\beta =\frac{\frac{1}{3}\left[\frac{1-\sin\varphi_{u,cr}}{1+\sin\varphi_{u,cr}}{\left(\frac{\sigma_{1}}{\sigma_{3}}\right)}_{cr}-1\right]}{\frac{\sigma_{1}}{p}\left(1-\frac{1-\sin\varphi_{u,cr}}{1+\sin\varphi_{u,cr}}\right)+\frac{1-\sin\varphi_{u,cr}}{1+\sin\varphi_{u,cr}}-1}$$

This derivation demonstrates that β is a physically based parameter determined jointly by the macroscopic critical state friction angle $\varphi_{cr}$ (which dictates the macroscopic critical stress ratio) and the microscopic critical inter-particle friction angle $\varphi_{u,cr}$. In practice, $\varphi_{u,cr}$ can be approximated as the intrinsic inter-particle friction angle. By combining this with the critical state stress ratio obtained from standard triaxial tests, the value of β can be uniquely determined.

(2) Dilatancy parameter $D_{0}$

The parameter $D_{0}$ characterizes the volumetric deformation tendency of the material at the onset of shearing. According to the dilatancy formulation within the proposed framework, for an initially isotropic material, the initial dilatancy ratio (where q=0) simplifies to $D_{ini}=D_{0}$. Consequently, $D_{0}$ is physically defined as the intrinsic initial dilatancy ratio for an isotropic granular assembly.

For initially anisotropic materials, the calibration of $D_{0}$ requires prior determination of the initial fabric state. The procedure involves the following steps: First, the fabric evolution parameter β and the initial fabric components ($F_{11}^{0},F_{22}^{0},F_{33}^{0}$) are determined, allowing for the calculation of the initial true deviatoric stress $q_{T}$ and the coefficient *C*. Subsequently, the evolution curve of the dilatancy ratio *D* versus the stress ratio q/p is plotted based on drained triaxial test data. The specific value of the initial dilatancy ratio $D_{ini}$ at the onset of shearing (i.e., as q/p→0) is extracted from this curve. Finally, by substituting these values into the general dilatancy equation (Equation (25)), the parameter $D_{0}$ can be uniquely back-calculated.

(3) Dilatancy parameter χ

The parameter χ serves as a pivotal plastic variable within the constitutive framework, governing the kinetics of microstructural evolution. Physically, it modulates the rate of coordination number evolution and defines the phase transformation threshold within the evolution law—essentially dictating the critical state where the dominance shifts between contact creation and contact disruption mechanisms.

**Figure 6:**
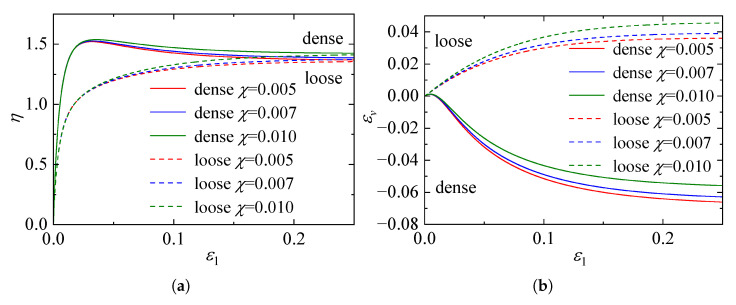
Effect of parameter χ on the mechanical response: (**a**) stress ratio–axial strain curves. (**b**) volumetric strain–axial strain curves.

Figure 6 presents the sensitivity of the macroscopic mechanical response to variations in the parameter χ (ranging from 0.005 to 0.010), as computed by the proposed constitutive model. A slight increase in the stress ratio is observed for both loose and dense sand as χ becomes larger, while the maximum dilatancy is slightly suppressed. Nevertheless, these differences remain minor in magnitude, and the predicted curves associated with the three parameter values display a nearly identical overall pattern. This result indicates that the macroscopic response of the model is only weakly dependent on this parameter, reflecting a generally low level of sensitivity. Besides, from a micromechanical perspective, χ should not be assigned excessively high values; otherwise, the evolution of the state variable λ becomes insignificant, failing to capture the dynamic restructuring of the contact network during shear. Balancing the macroscopic insensitivity with the necessity of preserving clear micromechanical evolution features, a range of 0.005 to 0.01 is recommended for parameter χ.

(4) Dilatancy parameter α

**Figure 7:**
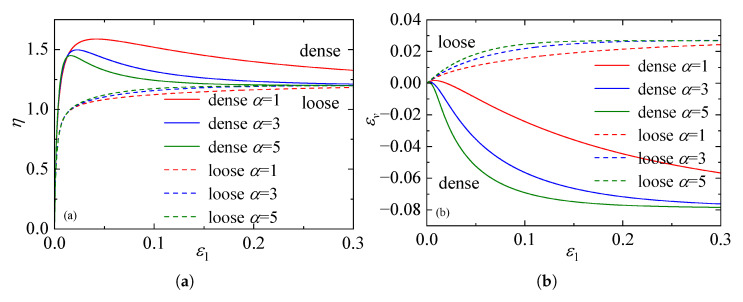
Effect of parameter α on the mechanical response: (**a**) stress ratio–axial strain curves. (**b**) volumetric strain–axial strain curves.

The parameter α serves as a plastic variable characterizing the sensitivity of dilatancy behavior to the material’s density state. Mathematically, as the exponent of the state parameter $e/e_{cr}$ within the contact evolution law, α governs the extent to which the void ratio constrains microstructural reorganization. Figure 7 presents the sensitivity analysis of the macroscopic mechanical response predicted by the proposed framework for both dense and loose sands subject to variations in α.

The results in Figure 7a,b reveal that α exerts a profound influence on the mechanical behavior. An increase in α amplifies the dependence of the structural evolution on the current density. Specifically, for dense specimens, a higher α value intensifies volumetric dilation (indicated by a deeper trough in the volumetric strain curve); however, this enhanced dilation is accompanied by increased energy dissipation, resulting in a marked reduction in the peak stress ratio. Conversely, for loose specimens, a larger α leads to pronounced volumetric contraction. Crucially, despite these variations in the evolutionary trajectory, the stress and volumetric strain curves for all α values converge at large strains. This confirms that α influences only the path towards the critical state without altering the asymptotic critical state values themselves. Given the complex coupling between density and dilatancy encapsulated by this parameter, direct experimental determination is challenging. Consequently, it is recommended to calibrate α via back-analysis or optimization methods against standard triaxial stress-strain and volumetric data.

(5) Strength parameter $m_{\varphi }$

The parameter $m_{\varphi }$ characterizes the dependence of the peak strength on the material density state. According to the proposed hardening law, the relationship between the peak friction angle $\varphi_{p}$ at the current void ratio *e* and the critical state friction angle $\varphi_{cr}$ is governed by:

(45) $$\tan\varphi_{p}={\left(\frac{e_{cr}}{e}\right)}^{m_{\varphi }}\tan\varphi_{cr}$$

where *e* and $e_{cr}$ denote the current void ratio and the corresponding critical state void ratio, respectively. For calibration, the peak void ratio $e_{p}$, peak friction angle $\varphi_{p}$, and critical state friction angle $\varphi_{cr}$ are directly obtained from standard triaxial compression tests. The critical state void ratio $e_{cr}$ is determined via the critical state line. Consequently, rearranging the equation yields the explicit solution for $m_{\varphi }$:

(46) $$m_{\varphi }=\frac{\ln\left(\tan\varphi_{p}\right)-\ln\left(\tan\varphi_{cr}\right)}{\ln e_{cr}-\ln e_{p}}$$

(6) Strength parameter *m*

The parameter *m* defines the orientation of the microscopic failure plane (or potential slip plane) relative to the true stress field, and is determined by combining data from triaxial compression (TC) and triaxial extension (TE) tests. Based on the critical friction angles obtained from these stress paths, the corresponding critical true stress ratios, $k_{T13}^{TC}$ and $k_{T13}^{TE}$, are calculated.

Within the proposed framework, the hardening parameter $H_{T}$ at the critical state is expressed as a function of the true stress ratios. The formulations for the TC and TE paths are given, respectively, by:

(47) $$\begin{array}{c} H_{T}^{TC}=\frac{\sqrt{{\left(k_{T13}^{TC}-k_{T13}^{TC}\right)}^{2}+{\left(k_{T23}^{TC}-1\right)}^{2}{\left(k_{T13}^{TC}\right)}^{m}+{\left(k_{T13}^{TC}-1\right)}^{2}{\left(k_{T23}^{TC}\right)}^{m}}}{{\left(k_{T13}^{TC}\right)}^{1-\frac{m}{2}}{\left(k_{T23}^{TC}\right)}^{\frac{m}{2}}+{\left(k_{T13}^{TC}\right)}^{\frac{m}{2}}{\left(k_{T23}^{TC}\right)}^{1-\frac{m}{2}}+{\left(k_{T13}^{TC}\right)}^{\frac{m}{2}}{\left(k_{T23}^{TC}\right)}^{\frac{m}{2}}} \end{array}$$

(48) $$\begin{array}{c} H_{T}^{TE}=\frac{\sqrt{{\left(k_{T13}^{TE}-k_{T13}^{TE}\right)}^{2}+{\left(k_{T23}^{TE}-1\right)}^{2}{\left(k_{T13}^{TE}\right)}^{m}+{\left(k_{T13}^{TE}-1\right)}^{2}{\left(k_{T23}^{TE}\right)}^{m}}}{{\left(k_{T13}^{TE}\right)}^{1-\frac{m}{2}}{\left(k_{T23}^{TE}\right)}^{\frac{m}{2}}+{\left(k_{T13}^{TE}\right)}^{\frac{m}{2}}{\left(k_{T23}^{TE}\right)}^{1-\frac{m}{2}}+{\left(k_{T13}^{TE}\right)}^{\frac{m}{2}}{\left(k_{T23}^{TE}\right)}^{\frac{m}{2}}} \end{array}$$

Invoking the uniqueness of the critical state, the hardening condition implies $H_{T}^{TC}\;=\;H_{T}^{TE}$. By substituting the experimental values into these expressions, the strength parameter *m* can be uniquely determined.

### 4.2. Analysis of Strength Anisotropy Under Varying Loading Directions

**Figure 8:**
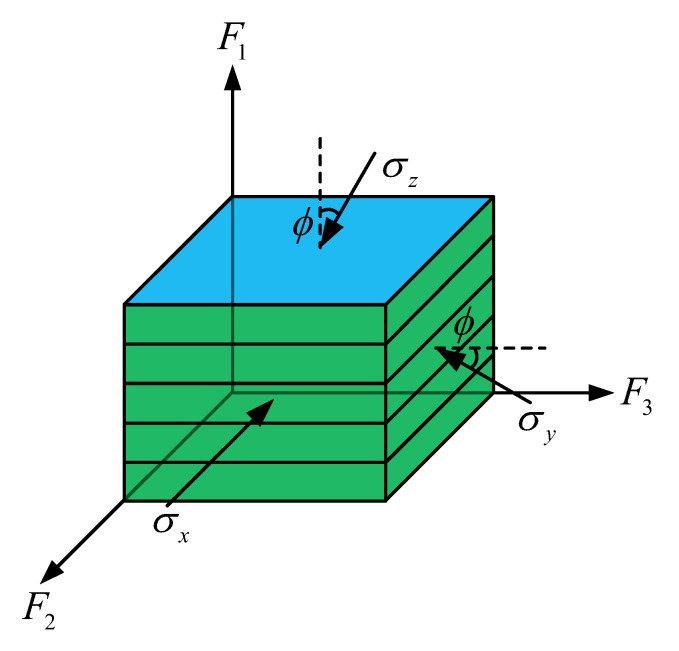
Schematic representation of the loading direction and the principal axes of fabric.

Natural soil deposits frequently exhibit pronounced inherent anisotropy. To investigate the evolution of material strength under varying fabric states, this section conducts model analyses on soils with initial transversely isotropic and orthotropic fabrics. To quantify the effect of loading direction, the angle ϕ between the major principal stress axis and the major principal axis of the initial fabric (i.e., the normal to the bedding plane) is varied across 0°, 30°, 45°, 60°, and 90°. The geometric relationship between the loading direction and the fabric principal axes is schematically illustrated in Figure 8.

**Figure 9:**
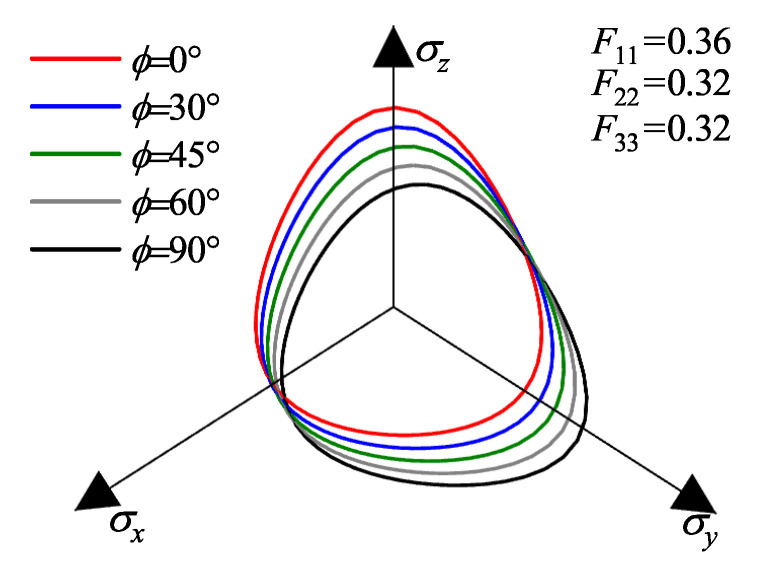
Strength envelopes in the π-plane for an initially transversely isotropic material under various loading directions.

**Figure 10:**
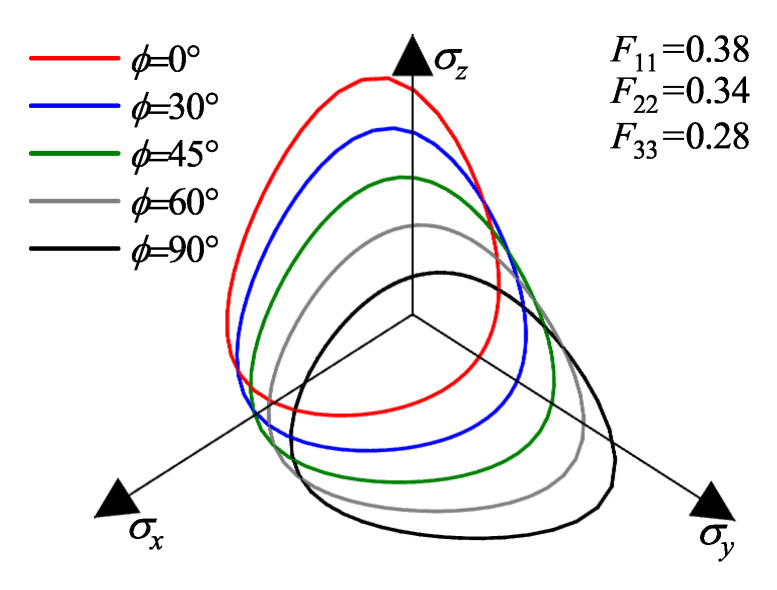
Strength envelopes in the π-plane for an initially orthotropic material under various loading directions.

Figure 9 and Figure 10 present the evolution of failure envelopes in the principal stress space for transversely isotropic and orthotropic materials, respectively, as calculated by the proposed strength criterion under different loading angles ϕ.

For transversely isotropic material (Figure 9), at ϕ=0°, where the loading axis coincides with the normal to the bedding plane, the failure envelope exhibits strict axial symmetry about the $\sigma_{z}$-axis, attributable to the equality of the lateral fabric components ($F_{22}=F_{33}$). The envelope extends furthest along the $\sigma_{z}$-axis (loading perpendicular to bedding) compared to the $\sigma_{x}$ and $\sigma_{y}$ axes (loading parallel to bedding), corroborating the established experimental observation that shear strength is maximized perpendicular to the depositional plane [39,40]. As ϕ increases (representing a rotation of the principal stress axes about the $\sigma_{x}$-axis), the failure envelope undergoes distinct distortion and translation: the strength intercept along the $\sigma_{z}$-axis decreases monotonically, while that along the $\sigma_{y}$-axis increases. This geometrically captures the realignment of the material’s dominant resistance direction from the *z*-axis to the *y*-axis as the loading direction rotates. Notably, the intercepts along the $\sigma_{x}$-axis remain invariant across all loading angles. This invariance arises because the rotation occurs within the y−z plane; consequently, the fabric component along the axis of rotation (*x*-direction) remains constant, resulting in a constant macroscopic strength in that direction.

For the orthotropic material (Figure 10), the inequality of the three orthogonal fabric components ($F_{11}>F_{22}>F_{33}$) results in a failure envelope that lacks axial symmetry even at ϕ=0°. Specifically, the envelope extends further along the $\sigma_{x}$-axis than the $\sigma_{y}$-axis. This asymmetry is consistent with the microstructural condition $F_{22}>F_{33}$, indicating that the intermediate fabric component exerts a governing influence on the shape of the macroscopic failure surface. With increasing ϕ, the evolution of the envelope mirrors the trend observed in the transversely isotropic case, with the dominant strength direction shifting from the $\sigma_{z}$-axis to the $\sigma_{y}$-axis. However, constrained by the initial orthotropic fabric, the morphological evolution of the envelope is more complex. Nevertheless, the constancy of strength along the $\sigma_{x}$-axis persists, further confirming the model’s capability to accurately capture the control of individual fabric components on directional strength.

## 5. Comparisons with Experimental Data and Numerical Simulations

To verify the validity and predictive capability of the proposed constitutive model, the model predictions are compared with results obtained from both laboratory experiments and Discrete Element Method (DEM) simulations. The calibrated model parameters employed for these validation exercises are summarized in Table 2. It is noted that these parameters are treated as deterministic material constants for each specific type of sand. The basic physical parameters (elastic and critical state parameters) were directly determined from the reported experimental data, while the micro-evolution parameters were calibrated via back-analysis within physically constrained ranges. Unless otherwise specified, all experimental and numerical validation cases presented in this section correspond to fully drained conditions conducted under quasi-static loading rates, ensuring that no excess pore pressure or inertial effects are involved.

### 5.1. Validation Against Drained Triaxial Tests on Ottawa Sand

**Figure 11:**
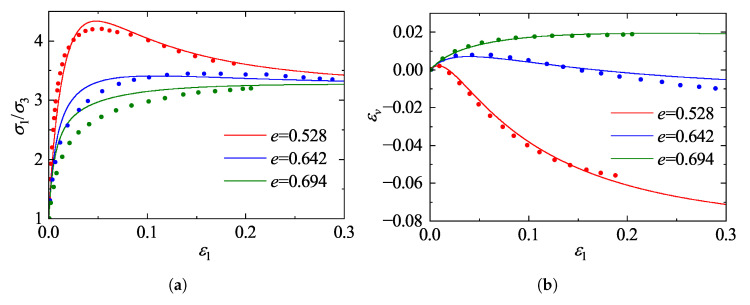
Comparison between model predictions and experimental data for Ottawa sand [41] at a confining pressure of 200 kPa: (**a**) stress ratio; (**b**) volumetric strain.

**Figure 12:**
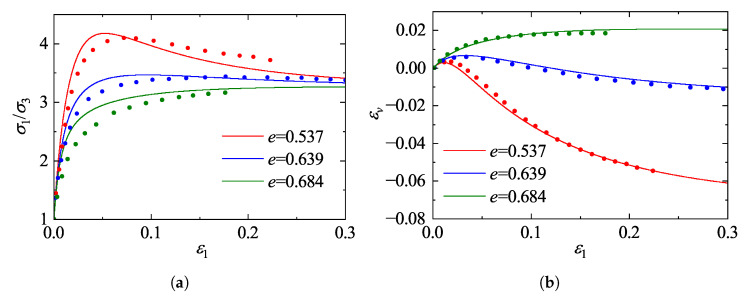
Comparison between model predictions and experimental data for Ottawa sand [41] at a confining pressure of 500 kPa: (**a**) stress ratio; (**b**) volumetric strain.

**Figure 13:**
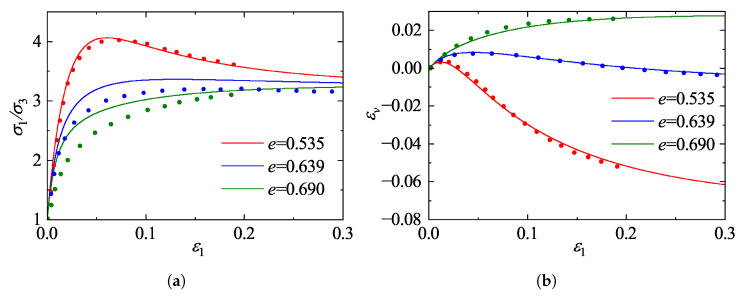
Comparison between model predictions and experimental data for Ottawa sand [41] at a confining pressure of 800 kPa: (**a**) stress ratio; (**b**) volumetric strain.

The predictive capability of the proposed framework is first evaluated against the comprehensive experimental dataset on Ottawa sand reported by Regier [41]. The experimental program consisted of standard drained conventional triaxial compression tests conducted on cylindrical specimens. These tests were performed under quasi-static, displacement-controlled conditions, ensuring full drainage and the elimination of inertial effects throughout the loading process. To cover a wide range of material states, the specimens were prepared at various initial void ratios representing loose, medium-dense, and dense states, and were isotropically consolidated to initial confining pressures of $\sigma_{3}=200,\;500,$ and 800 kPa, respectively. The model predictions were generated using the calibrated constitutive parameters listed in Table 2. Figure 11, Figure 12 and Figure 13 illustrate the comparisons between model predictions and experimental data. The stress-strain responses reveal a strong dependence on the initial state: dense specimens exhibit distinct strain-softening behavior, while loose specimens display characteristic strain-hardening. The model accurately captures the peak strength and the subsequent softening trajectory of the dense sand. Notably, as the confining pressure increases from 200 kPa to 800 kPa, the peak stress ratio decreases and the extent of softening diminishes, indicating a transition towards more ductile failure. The proposed model effectively reproduces this stress-level dependency of the strength characteristics.

Regarding volumetric deformation, the model successfully replicates the phase transformation behavior (contraction followed by dilation) observed in dense sand, as well as the continuous contraction of loose sand. A comparison across the different confining pressures highlights the suppression of dilatancy by higher stress levels; specifically, the magnitude of dilation in dense sand at 800 kPa is markedly reduced compared to that at 200 kPa. By incorporating state-dependent dilatancy formulations, the model accurately captures this pressure-induced suppression of dilatancy. Although the initial shear stiffness for loose sand is slightly overestimated in the small-strain regime, the overall predictions demonstrate a high degree of agreement with the experimental measurements in terms of peak strength, critical state approach, and volumetric evolution, confirming the robustness of the model in describing granular mechanics under conventional triaxial paths.

### 5.2. Validation Against True Triaxial Numerical Tests

To evaluate the predictive capability of the proposed framework under complex three-dimensional stress paths, the model predictions are compared with the true triaxial DEM simulation results reported by Jiang et al. [42]. In their study, numerical specimens were prepared at two distinct initial densities, corresponding to initial void ratios of 0.67 and 0.82, and were subsequently isotropically consolidated to a mean effective stress of 100 kPa. Following consolidation, true triaxial shear tests were performed under stress-controlled conditions. To systematically investigate the influence of the intermediate principal stress, the simulations covered the full range of the intermediate principal stress ratio, $b=\left(\sigma_{2}-\sigma_{3}\right)/\left(\sigma_{1}-\sigma_{3}\right)$, with specific values set to 0.0, 0.2, 0.4, 0.6, 0.8, and 1.0, respectively. The model parameters were calibrated directly against this DEM dataset, and their values are listed in Table 2.

**Figure 14:**
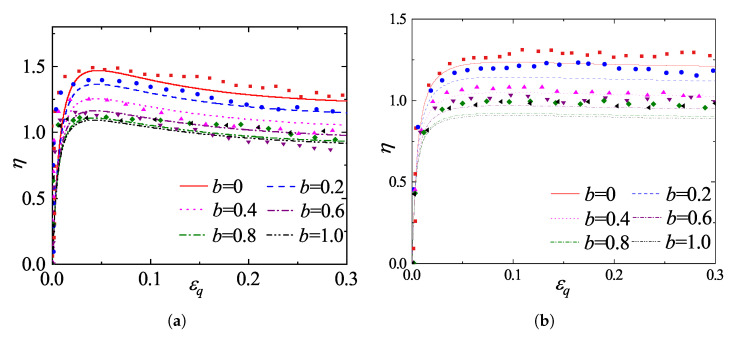
Comparison between model predictions and DEM simulations [42] for different intermediate principal stress ratios *b*: (**a**) dense sample (e=0.67); (**b**) loose sample (e=0.82).

Figure 14 illustrates the comparisons of stress ratio (η=q/p)-strain responses for specimens with different initial void ratios sheared under a constant confining pressure of 100 kPa at varying intermediate principal stress ratios *b*. As depicted in Figure 14, both the initial void ratio and the *b*-value significantly influence the mechanical response. The denser specimens (e=0.67, Figure 14a) exhibit distinct strain-softening behavior, whereas the looser specimens (e=0.82, Figure 14b) display predominant strain-hardening characteristics. The model accurately captures the dependency of strength on the intermediate principal stress; specifically, both the peak and critical state stress ratios exhibit a decreasing trend as the *b*-value increases from 0 to 1. Notably, for b>0.6, the stress-strain curves tend to converge, suggesting that the internal friction angle remains essentially constant in this range, a phenomenon consistent with previous experimental findings [44]. Overall, the close agreement between the model predictions and the DEM data for both dense and loose states demonstrates the robustness of the proposed model in describing the mechanical behavior of granular materials under general three-dimensional stress conditions.

### 5.3. Validation Against Effects of Inherent Anisotropy on Toyoura Sand

The mechanical response of sand depends not only on initial void ratio and stress path but fundamentally on the initial particle arrangement, termed inherent fabric anisotropy. This section examines the model’s performance under different initial fabric anisotropies by comparing simulations against experimental data for Toyoura sand from Lam and Tatsuoka [43]. The experiments employed dense specimens prepared via air-pluviation to induce a distinct transversely isotropic fabric. The testing program consisted of drained triaxial compression tests on samples isotropically consolidated to $\sigma_{3}=98$ kPa, conducted under quasi-static conditions (axial strain rate of 0.25%/min) with lubricated ends to minimize boundary friction. The validation specifically targets the anisotropic strength and dilatancy characteristics by simulating loading paths with bedding plane angles of ϕ=0°, 30°, 60°, and 90°. The model parameters used in these simulations are given in Table 2.

**Figure 15:**
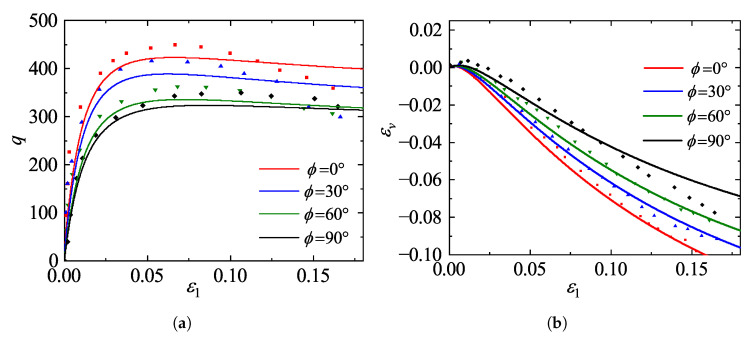
Measured [43] and predicted responses for specimens with various initial deposition angles: (**a**) deviatoric stress versus axial strain; (**b**) volumetric strain versus axial strain.

Figure 15 presents the comparisons of deviatoric stress *q* and volumetric strain $\varepsilon_{v}$ versus axial strain $\varepsilon_{1}$ for specimens with different initial deposition angles (ϕ = 0°, 30°, 60°, and 90°). As illustrated in Figure 15a, the deposition angle exerts a profound influence on the strength characteristics. The specimen with ϕ=0° (where the major principal stress is perpendicular to the bedding plane) exhibits the highest peak strength accompanied by pronounced strain softening. As the deposition angle increases, the peak strength decreases monotonically, and the mechanical response progressively transitions from strain softening to strain hardening. Correspondingly, the volumetric strain curves in Figure 15b reveal that dilatancy is increasingly suppressed with higher deposition angles, leading to greater volumetric contraction, which highlights the constraint imposed by the anisotropic fabric on volumetric deformation. The results show that the proposed model effectively reproduces the changes in mechanical behavior resulting from the anisotropy of sand.

Besides, it is particularly noteworthy that the model successfully reproduces the non-linear reduction in strength observed in experiments. Specifically, the rate of strength degradation decreases significantly as the deposition angle increases from 60° to 90°, which is consistent with earlier observations [45]. However, a discrepancy exists between the predicted and experimental residual strengths at large strains. This difference may be attributed to the current fabric evolution law, which does not explicitly account for the non-coaxial evolution between the principal stress axes and the fabric axes. Incorporating this non-coaxiality will be an important direction for future development of the model.

### 5.4. Validation Against Stress–Dilatancy Relationships in True Triaxial DEM Simulations

To further verify the accuracy and generality of the proposed model, particularly its performance in describing volumetric deformation under complex stress states, the model predictions were compared with the discrete element numerical simulation results reported by Wang et al. [46]. In their study, a series of true triaxial drained tests were conducted on granular assemblies using the DEM to investigate the evolution of shear strength and dilatancy. The simulations were performed under a constant mean effective stress of p=100 kPa, with the intermediate principal stress ratio *b* varying from 0 to 1.0. The initial void ratio of the dense specimen was set to $e_{0}=0.658$. For the model calibration in this section, the specific dilatancy parameters were identified as χ=0.01, α=3.2, and $D_{0}=0.375$, while other basic material parameters were kept consistent with the properties of the granular assembly in the simulation.

**Figure 16:**
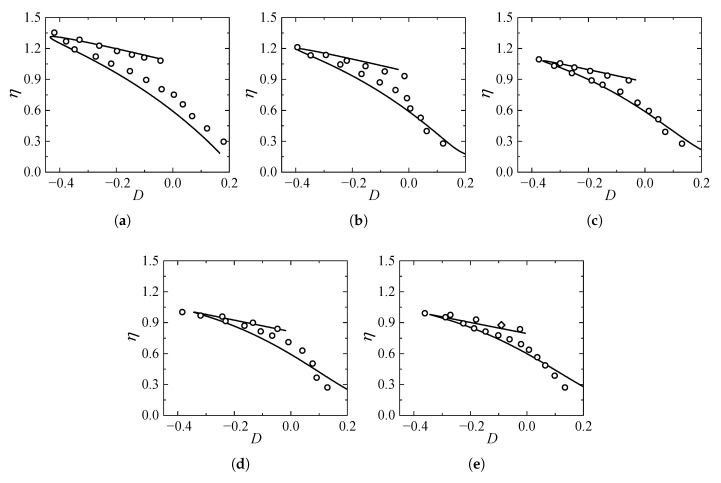
Comparison of stress–dilatancy relationships between model predictions and DEM simulations [46] under varying intermediate principal stress ratios *b*: (**a**) b=0; (**b**) b=0.25; (**c**) b=0.5; (**d**) b=0.75; (**e**) b=1.0.

Figure 16 illustrates the comparison between the model-predicted stress–dilatancy relationships (D−η) and the DEM simulation results under varying *b* values. As observed in the figure, the proposed model effectively captures the dependency of the dilatancy behavior on the intermediate principal stress. Specifically, the theoretical calculations successfully describe the trend where both the peak dilatancy ratio and the peak stress ratio decrease noticeably as the coefficient *b* increases from 0 to 1. Additionally, the model predictions exhibit excellent agreement with the simulation data throughout the shearing process, encompassing both the pre-peak hardening and post-peak softening regimes. Notably, the position of the phase transformation point (D=0), which marks the critical transition from volumetric contraction to dilation in dense sand, is accurately predicted and aligns closely with the DEM results. Overall, the proposed energy-based stress–dilatancy equation demonstrates a robust capability to reproduce the complex dilatancy states of granular materials under general three-dimensional stress conditions.

## 6. Conclusions

Addressing the pronounced mechanical anisotropy inherent in granular soils, this study derives the expression for the true stress tensor from a micromechanical perspective. By integrating this concept with critical state theory and the mechanism of coordination number evolution, an elasto-plastic constitutive model is established to unify the description of anisotropic evolution in sand. Through systematic parametric analysis and comprehensive validation against laboratory experiments and DEM simulations, the following conclusions are drawn:

(1) The macroscopic nominal stress represents a coupling of mechanical resistance and microstructural fabric, whereas the true stress tensor is defined as the product of the stress tensor and the inverse of the fabric tensor. Physically, the true stress tensor mathematically filters out the geometric non-uniformity of particle arrangements, thereby recovering the authentic stress intensity acting on the granular skeleton. Serving as the fundamental internal variable governing yield and failure, the true stress tensor achieves a geometric decoupling between macroscopic response and microstructural features, providing a robust physical basis for developing constitutive models capable of accurately representing the anisotropic behavior of sands.

(2) The proposed constitutive framework incorporates parameters with distinct physical interpretations, all of which can be calibrated via standard laboratory tests. Specifically, the strength parameter $m_{\phi }$ quantifies the dependency of the sliding friction angle on soil density, thereby governing the peak shear strength, while the parameter *m* relates to the direction of inter-particle sliding and can be determined from triaxial compression and extension tests. The parameter β governs the kinetics of fabric evolution and is intrinsically linked to the inter-particle friction coefficient. $D_{0}$ defines the initial dilatancy ratio, whereas the critical parameter χ governs the rate of coordination number evolution, serving as a fundamental link between micro-topological changes and macroscopic hardening. Furthermore, the parameter α elucidates the regulation of dilatancy by the density state; a higher α value intensifies both the dilatancy in dense sands and the contraction in loose sands, effectively quantifying the degradation of macroscopic peak strength induced by pronounced dilatancy.

(3) The model’s predictive capability is validated through comprehensive comparisons with drained triaxial tests on Ottawa sand, true triaxial DEM simulations, and anisotropic experiments on Toyoura sand. Under conventional loading, the model reproduces state-dependent responses, accurately capturing the strain-softening and phase transformation in dense specimens, as well as the strain-hardening and contractive behavior in loose deposits. It also describes the suppression of dilatancy under elevated confining pressures, reflecting the transition toward ductile failure modes. Under true triaxial stress paths, the framework demonstrates strong predictive performance; it quantifies the reduction in peak strength induced by increasing intermediate principal stress ratios (*b*-value) and captures the influence of inherent fabric anisotropy. Specifically, the model reproduces the transition from brittle to ductile response and the non-linear strength degradation associated with increasing deposition angles.

Despite the satisfactory predictive capability demonstrated above, certain limitations remain in the current framework. The theoretical derivation of the true stress tensor is presently based on the idealization of monodisperse spherical particles. However, natural granular materials, such as the sands analyzed in this study, inevitably possess angular morphologies and polydisperse grain size distributions. Although the use of an average radius provides a reasonable approximation, this geometric simplification overlooks the interlocking effects arising from particle angularity and the influence of gradation on the coordination number evolution, which contributes to the minor discrepancies observed between the model simulations and experimental results. Consequently, future research will focus on extending the micromechanical framework to explicitly incorporate statistical descriptors for particle shape and size distribution, potentially through the introduction of a shape tensor or grading-dependent state parameters. In parallel, to bridge the gap between theoretical micromechanics and engineering practice, efforts are underway to implement this enhanced constitutive model into commercial Finite Element software, thereby enabling the analysis of boundary value problems in complex geotechnical infrastructure.

## Figures and Tables

**Table 1:** Summary of model parameters.

Parameter Group	Symbols
Elasticity parameters	$G_{0},c_{g},\mu$
Critical state parameters	$e_{cr0},n_{cr},h_{cr}$
Strength and hardening parameters	$m_{\varphi },m,\varphi_{cr}$
Dilatancy parameters	$D_{0},\alpha ,\chi$
Fabric evolution parameters	$\beta ,F_{11}^{0},F_{22}^{0},F_{33}^{0}$

**Table 2:** Calibrated model parameters used for validation.

Parameter Type	Parameter	Ottawa Sand [41]	DEM Simulation [42]	Toyoura Sand [43]
Elasticity parameters	$G_{0}$ (MPa)	2.75	0.5	2.5
	$c_{g}$	2.17	2.97	2.17
	μ	0.29	0.29	0.29
Critical state parameters	$e_{cr0}$	0.74	0.86	1.12
	$h_{cr}$ (MPa)	2867	71.81	17
	$n_{cr}$	0.232	0.74	0.82
Yield surface parameters	*m*	0.581	0.581	0.5
Hardening parameters	$m_{\phi }$	2.3	2	0.8
	$\varphi_{cr}$ (°)	32	30	30
Dilatancy parameters	χ	0.007	0.007	0.005
	α	1.5	2.0	2.5
	$D_{0}$	0.74	0.7	0.8
Fabric parameters	$F_{11}^{0}$	1/3	0.34	0.36
	$F_{22}^{0}$	1/3	0.33	0.32
	$F_{33}^{0}$	1/3	0.33	0.32
	β	0.1	0.1	0.15

## Data Availability

The original contributions presented in this study are included in the article. Further inquiries can be directed to the corresponding author.
